# Barriers and Facilitators of Telemedicine Among Physicians at a University Hospital

**DOI:** 10.7759/cureus.45078

**Published:** 2023-09-12

**Authors:** Syed Habib, Khaled B Alsulaim, Osama A Mobeirek, Abdullah M Alsaeed, Fahad A Albawardi, Yazeed K Alqahtani, Abdulrhman A Alsuhaibany

**Affiliations:** 1 Physiology, King Saud University, Riyadh, SAU; 2 College of Medicine, King Saud University, Riyadh, SAU

**Keywords:** communication, virtual consultation, prevalence, facilitators, barriers, telemedicine

## Abstract

Background and aims

This study explored the perceived barriers and facilitators to the implementation of telemedicine among physicians and estimated and compared the prevalence of telemedicine use among physicians before and during coronavirus disease 2019 (COVID-19).

Methods

This cross-sectional study was conducted at King Saud University in Riyadh. A convenience sample of 163 physicians working at King Khalid University Hospital (KKUH) completed an online survey. Demographic data, patterns of use, and perceived barriers and facilitators of telemedicine were collected using a 5-point Likert scale.

Results

Our research showed that 61.3% (n = 100) of the physicians surveyed had used telemedicine in their careers. The prevalence of telemedicine before the onset of COVID-19 was 18.4%, whereas during COVID-19, it increased to 59.5%, which is an increase of 330% (P < .001). Most of the respondents (50.9%; n = 83) used it weekly (27%) or occasionally (23.9%). The most prevalent perceived barrier was technical difficulties (68.7%; n = 112), and the most prevalent perceived facilitator was that telemedicine can reduce unnecessary face-to-face appointments (86.5%; n = 141).

Conclusion

The use of telemedicine to provide health care is on the rise, especially in the case of emergencies. Different specialties face different facilitators and barriers, and the potential of telemedicine implementation depends on the work environment and the needs of the specialty. Several obstacles need to be overcome before telemedicine becomes a consistently used method for providing health care.

## Introduction

Telemedicine is the use of electronic information and communications technology to provide healthcare services to patients from a distance [1]. Telemedicine is a broad term encompassing synchronous, asynchronous, and remote patient monitoring. Synchronous telemedicine refers to virtual visits that allow physicians to interact and communicate with patients in real time using different modalities, such as phone calls, video calls, and text messaging. Asynchronous telemedicine, also known as “store and forward,” enables physicians or patients to access medical histories, pathology results, and even images, which can then be sent to a specialist to further a patient’s treatment. Remote patient monitoring involves the use of technological methods by healthcare workers to follow up on patients’ statuses [2]. The coronavirus disease 2019 (COVID-19) pandemic accelerated the implementation and innovation of telemedicine because it became essential to public health and safety rather than an optional approach to the provision of health care. Social distancing requirements and stay-at-home orders converted face-to-face consultations into virtual consultations. A study conducted in the US found a 683% increase in visits to telemedicine clinics during the COVID-19 pandemic [3]. Moreover, another study estimated a considerable increase in the use of telemedicine during COVID-19, with nearly 93% of care being delivered virtually, whereas a few weeks prior, telemedicine accounted for under 5% of patient visits [4]. As indicated in the literature, facilitators of telemedicine implementation have found that it can reduce expenses significantly and is a very cost-effective way to care for patients from a distance [5-7]. For example, a teleneurology study done in 2018 established that the use of telemedicine saved each patient approximately US$70 and two hours of driving [8]. Telemedicine can also reduce the number of unnecessary follow-up visits, which saves the time of both doctors and patients. Telemedicine has also been found to increase healthcare access [9,10]. Barriers to telemedicine implementation include clinicians having difficulty detecting non-verbal cues while interacting with patients and the lack of physical examinations, which might decrease diagnostic accuracy [11]. The privacy of patients using telemedicine and technical difficulties encountered by both patients and physicians are also barriers to telemedicine implementation [12]. Many methods used in telemedicine enable healthcare professionals to communicate with their patients effectively. These methods include phone calls, video calls, and text messages, with phone calls being the most common [13]. Telemedicine has played a major role in providing essential health care during the COVID-19 pandemic, creating the need for more extensive research into this emerging field, especially at the regional and local levels, given the different challenges that physicians may face in different regions and countries. A study conducted in Taif discussed the advantages and disadvantages of telemedicine [5], and another was conducted in the Eastern Province [10]. However, to the best of our knowledge, no study has researched the barriers and facilitators of telemedicine implementation among physicians in Riyadh. Therefore, this study aims to explore the perceived facilitators and barriers to the implementation of telemedicine among physicians at King Khalid University Hospital (KKUH) and to estimate and compare the prevalence of telemedicine use among physicians at KKUH before and during COVID-19. We hypothesize that telemedicine implementation has increased since the beginning of the COVID-19 pandemic.

## Materials and methods

A cross-sectional study design was used to achieve the objectives of the study, which was carried out at KKUH in 2021. This hospital is a tertiary care center with multiple specialty departments. It is a teaching hospital that belongs to the College of Medicine at King Saud University in Riyadh, Saudi Arabia.

A convenience sampling technique was chosen for this study. To determine the sample size, the following formula was used: n = (Zα + Zβ)2 * (p1(1 - p1) + p2(1 - p2)) / (p1 - p2)2, where p1 is the proportion (prevalence) of telemedicine use among physicians before the COVID-19 pandemic, and p2 is the proportion (prevalence) of telemedicine use among physicians during the COVID-19 pandemic. P1 was calculated to be 33.3% [10], and p2 was calculated to be 58.1% [6]. Thus, the sample size was calculated to be 160.

As for the distributions, most medical departments received an email invitation with a copy of the Institutional Review Board’s approval requesting that their physicians voluntarily participate in the study to reduce selection bias and face-to-face interactions to ensure a greater sample. All physicians who responded working in the hospital from July 2021 to October 2021 were included, regardless of their position level, specialty, or experience with telemedicine. Other health practitioners were excluded from the study.

The study variables were age, gender, professional position, specialty, nationality, and pattern of use of telemedicine. The study outcomes were perceived barriers and facilitators, prevalence of use of telemedicine before COVID-19, and prevalence of use of telemedicine after COVID-19.

We conducted an extensive review of the literature to identify 13 barriers and facilitators to include in the study. We then chose specific statements to represent perceived barriers and facilitators to include in our questionnaire, so that we could compare the findings of our study to those reported in the literature.

The questionnaire was formulated by the researchers after conducting a literature review. The questionnaire included questions on the physicians’ demographic data, their experience and patterns with telemedicine utilization, and their opinions regarding barriers and facilitators to the implementation of telemedicine. The questionnaire was a close-ended, self-administered format written in English, and an electronic link was used for distribution. All items were compulsory to avoid missing data. A pilot study was conducted to estimate the time needed to complete the survey and to test logistics and comprehensibility.

The study followed all ethical considerations. The proposal was submitted to and approved by the Institutional Review Board of the College of Medicine at KSU. Data collection began after ethical approval and departmental permission were acquired. The physicians’ contributions were voluntary, and informed consent was obtained from all participants. The data were made anonymous and confidential.

Data analysis

The collected data were analyzed using IBM SPSS Statistics for Windows, Version 28 (Released 2021; IBM Corp., Armonk, New York, United States). The scores and number of responses were computed using Microsoft Excel (Microsoft Corporation, Redmond, USA). Descriptive statistics (frequencies and percentages) were used to describe the categorical data. Bivariate statistical analysis was carried out using a chi-squared test. A p-value of ≤ 0.05 indicated statistical significance. The main outcome measures were physician specialty, use, patterns of use, and views about barriers and facilitators to the implementation of telemedicine, which were measured using a 5-point Likert scale.

## Results

A total of 163 physicians participated in this study. All were working at KKUH at the time of the study. The vast majority of the study sample was Saudi physicians, and most (74.2%; n = 121) were 20-40 years old. The male-to-female ratio was 1.8:1. Consultant physicians accounted for about one-third of the sample at 33.7% (n = 55). The respondents worked in a variety of medical disciplines; internists and surgical subspecialists were among the most common respondents. The demographic characteristics of the respondents are shown in Table 1.

**Table 1:** Distribution of demographic characteristics of study participants (n = 163) *Other specialties (Pediatric, obstetrics and gynecology, radiology, anesthesia, dermatology, emergency, family medicine, intern, ophthalmology, and psychiatry)

Characteristics	No. (%)
Age	
(21–30)	84 (51.5)
(31–40)	37 (22.7)
(41–50)	22 (13.5)
(51–60)	15 (9.2)
(> 60)	5 (3.1)
Gender (Male)	105 (64.4)
Job Position	
Intern	47 (28.8)
Resident	38 (23.3)
Specialist	23 (14.1)
Consultant	55 (33.7)
Specialty	
Internal medicine	46 (28.2)
Surgery	29 (17.8)
Others*	88 (54)
Nationality (Saudi)	142 (87.1)
* Other specialties (Pediatric, obstetrics and gynecology, radiology, anesthesia, dermatology, emergency, family medicine, intern, ophthalmology, and psychiatry)	

**Figure 1:**
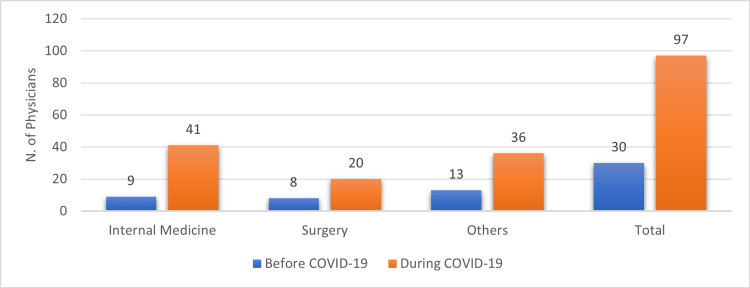
Comparison of telemedicine utilization among physicians before and during COVID-19

Table 2 shows that more than half (61.3%; n = 100) of the physicians in this study had used telemedicine in the course of their careers. The specialty that used telemedicine the most was internal medicine (89.1%; n = 41). The use of telemedicine during COVID-19 tripled from 18.4% (n = 30) before the pandemic to 59.5% (n = 97) during the pandemic as illustrated in Figure 1. Internal medicine respondents showed the highest increase in telemedicine use during COVID-19 at 78% (n = 32). The most common methods for providing telemedicine services were phone calls (57.7%; n = 94), text messages (19.6%; n = 32), and video consultations (8.6%; n = 14). Email was the least-used method among all physicians at 8% (n = 13). Internal medicine physicians used all methods the most, except video, which was more frequently used by other specialties (24.3%; n = 9). As for the frequency of telemedicine use, 44 (27%) physicians used it weekly, 39 (23.9%) used it occasionally, and 17 (10.4%) used it daily. Most of the internal medicine respondents used telemedicine weekly at 56.1% (n = 23), while surgeons used it at 45.5% (n = 10), and other specialists used telemedicine occasionally at 40.5% (n = 15). Surgery represented the highest proportion of telemedicine use daily at 22.7% (n = 5).

**Table 2:** Distribution and comparison of prevalence and patterns of telemedicine utilization across the three specialties of physicians (n = 163) Data are presented as number and percentages of participants n (%). Data were compared using chi-squared tests.

Variables	Prevalence of telemedicine		Time of usage of telemedicine		Methods of utilization				Frequency of usage of telemedicine		
	Yes	Never	Before COVID-19	During COVID-19	Phone calls	Video calls	Text messages	Emails	Daily	Weekly	Occasionally
Internal medicine	41 (25.2)	5 (3.1)	9 (5.5)	41 (25.1)	41 (25.1)	1 (0.6)	14 (8.6)	5 (3.1)	4 (2.45)	23 (14.1)	14 (8.6)
Surgery	22 (13.5)	7 (4.3)	8 (4.9)	20 (12.3)	20 (12.3)	4 (2.5)	11 (6.7)	6 (3.7)	5 (3.1)	7 (4.3)	10 (6.1)
Others	37 (22.7)	51 (31.3)	13 (8)	36 (22.1)	33 (20)	9 (5.5)	7 (4.3)	4 (2.5)	5 (3.1)	14 (8.6)	15 (9.2)
Total	100 (61.3)	63 (38.7)	30 (18.4)	97 (59.5)	94 (57.7)	14 (8.6)	32 (19.6)	13 (8)	17 (10.4)	44 (27)	39 (23.9)
p-value	< .001		0.295	< .001	< .001	0.509	< .001	0.03	< .001		

This study experienced a variable response in terms of agreement regarding the perceived barriers and facilitators of telemedicine implementation, with significant p-values for all items on the 5-point Likert scale. The most agreed-upon barrier was technical difficulties (68.7%; n = 112) (Table 3). However, patient’s privacy had the highest disagreement at 47.3% (n = 77). The barrier agreement responses tended to be lower in percentage compared to the facilitator responses; the overall percentages for agreement regarding facilitators and barriers were 69.11% and 50.94%, respectively. When correlated with specialties, we identified that lack of training was the most frequent barrier among internal medicine physicians at 71.8% (n = 33), while surgeons chose outdated hardware as their top barrier at 65.5% (n = 19). Regarding the facilitators, enhancement in the accessibility to health care had a prevailing consensus of 80.4% (n = 131) (Table 4). However, lowering unnecessary face-to-face appointments was the most reported facilitator among internists at 97.5% (n = 45).

**Table 3:** Distribution and comparison of responses toward perceived barriers to telemedicine implementation (n = 163) Data were compared using chi-squared tests.

Barriers	Strongly disagree, n (%)	Disagree, n (%)	Neutral, n (%)	Agree, n (%)	Strongly agree, n (%)	P-value
Telemedicine diagnosis is unreliable due to a lack of physical examination.	5 (3.1)	27 (16.6)	61 (37.4)	41 (25.2)	29 (17.8)	< 0.001
There is a lack of training in the use of telemedicine for physicians.	6 (3.7)	8 (4.9)	44 (27.0)	47 (28.8)	58 (35.6)	< 0.001
Patient’s privacy is a barrier to the implementation of telemedicine.	41 (25.2)	36 (22.1)	49 (30.1)	21 (12.9)	16 (9.8)	< 0.001
Physicians are resistant to changing from face-to-face consultations to virtual consultations.	11 (6.7)	32 (19.6)	62 (38.0)	35 (21.5)	23 (14.1)	< 0.001
Technical difficulties are a barrier to telemedicine implementation.	4 (2.5)	14 (8.6)	33 (20.2)	62 (38.0)	50 (30.7)	< 0.001
Outdated hardware is a barrier to telemedicine.	6 (3.7)	16 (9.8)	43 (26.4)	35 (21.5)	63 (38.7)	< 0.001
Difficulty in detecting non-verbal cues is a barrier to telemedicine.	3 (1.8)	16 (9.8)	43 (26.4)	60 (36.8)	41 (25.2)	< 0.001

**Table 4:** Distribution and comparison of responses toward perceived facilitators of telemedicine implementation (n = 163) Data were compared using chi-squared tests.

Facilitators	Strongly disagree, n (%)	Disagree, n (%)	Neutral, n (%)	Agree, n (%)	Strongly agree, n (%)	P-value
Telemedicine can lower unnecessary patient’s face-to-face appointments	3 (1.8)	4 (2.5)	15 (9.2)	43 (26.4)	98 (60.1)	< 0.001
Telemedicine is easily understood and used by physicians .	5 (3.1)	25 (15.3)	44 (27.0)	43 (26.4)	46 (28.2)	< 0.001
Telemedicine is cost efficient for the hospital.	4 (2.5)	10 (6.1)	26 (16.0)	41 (25.2)	82 (50.3)	< 0.001
Telemedicine can increase patients’ understanding and adherence to treatment plans.	18 (11.0)	34 (20.9)	46 (28.2)	43 (26.4)	22 (13.5)	0.001
Telemedicine can ensure high standards of health care.	5 (3.1)	24 (14.7)	53 (32.5)	55 (33.7)	26 (16.0)	< 0.001
Telemedicine enhances the accessibility to health care.	5 (3.1)	6 (3.7)	21 (12.9)	64 (39.3)	67 (41.1)	< 0.001

## Discussion

The results showed that the most significant perceived barrier to telemedicine use was technical difficulties, and the most significant facilitator was lowering unnecessary patient face-to-face appointments. We also found that the prevalence of telemedicine use increased from 18.4% before COVID-19 to 59.5% during COVID-19. Therefore, the implications of telemedicine and its capacity to provide high-quality care are becoming more apparent as a result of the COVID-19 pandemic, and telemedicine services have become essential, especially in pandemics or for those who have limited access to health care.

Telemedicine use in our sample increased by 330% during COVID-19. Similarly, a study conducted in the US in 2020 showed a 683% increase in telemedicine and telehealth use after the onset of COVID-19.3 This shows the increasing trend in telemedicine usage and further solidifies its significance in the provision of healthcare services. Furthermore, we found that the most frequently used method for conducting telemedicine was phone calls. A similar result was found by Mubaraki et al. [5]. On the other hand, a national survey conducted in Egypt in 2022 found that mobile applications were the most commonly used telemedicine service [14]. Regarding the frequency of telemedicine usage, we found a variable response, where 44% of the physicians who used telemedicine used it weekly, and only 17% used it daily. In contrast to our findings, a nationwide study conducted in China in 2022 found that 74% of healthcare professionals used telemedicine once a week [15].

There are many barriers to the implementation of telemedicine, and one of the most frequently encountered barriers is the lack of physician training in telemedicine. We found that 64.4% of physicians agreed that there is a lack of training in the use of telemedicine, which reflects the results of a study on family medicine physicians, which reported that 54% of physicians indicated a lack of training [16]. Therefore, a lack of training is a significant contributing factor to the large number of technical difficulties faced by physicians. A study published in 2021 showed that most physicians (90%) agreed that patient's privacy is a major barrier to the implementation of telemedicine in healthcare systems [17]. This is inconsistent with our findings and a study aimed at the general population in Egypt, which found that only 22.7% of physicians and 21.9% of the public, respectively, considered privacy to be an issue [18]. The variation could be attributed to the fact that their study was conducted in 2016, and the fast evolution of internet security in recent years has reduced the possibility of potential privacy breaches altering physicians’ opinions on this matter. Furthermore, we found that nearly half of the physicians in our study believed that diagnosis through telemedicine is unreliable due to the lack of physical examinations. A similar study conducted in 2021 reported that physicians had difficulties establishing the correct diagnosis due to a lack of physical examination [5]. Regarding physicians’ resistance to change from face-to-face consultations to virtual consultations, we found that 35% of physicians had difficulties adapting to delivering care virtually, the reasoning behind which can be attributed to the rapid shift to telemedicine during COVID-19, in contrast to conventional hands-on health care.

A major aspect of our study viewed the facilitators of telemedicine applications at KKUH. One of the most common concerns regarding the use of telemedicine in health care worldwide rests on whether telemedicine can provide adequate standards of health care. A study in 2020 that discussed physicians’ attitudes toward telemedicine use in Saudi Arabia concluded that only 27.8% of licensed physicians consider telemedicine a good diagnostic tool compared to face-to-face consultations [6]. In contrast, our study showed that half of the physicians believed telemedicine can provide a high standard of health care. That being said, we think that the quality of care telemedicine provides depends on the nature of the medical field itself; therefore, specialties, such as radiology, that rely on patients’ visual data are more appropriate for telemedicine. As anticipated, the majority of participants concurred on the commonly sensed facilitators of telemedicine in decreasing unnecessary face-to-face appointments, ultimately resulting in time and cost savings for both patients and healthcare institutions. This suggests that telemedicine can play a significant role in reducing the number of unnecessary hospital visits and effectively ensuring that patients in need of urgent care receive it faster, which we trust would ultimately solve the significant issue of providing emergency care that affects the whole world. Interestingly, physicians in our study expressed a low level of agreement regarding telemedicine’s role in increasing patients’ understanding and adherence to treatment plans, whereas another study [19] that focused on the role of telemedicine in diabetic patients’ care concluded that the application of telemedicine services remarkably increased patients’ adherence to the management plan, illustrating the huge impact telemedicine has on patients’ compliance. Our findings revealed that 80% of the physicians accepted that telemedicine improved access to care and that it played an integral role in response to the rapid transition to telemedicine during the COVID-19 crisis. Similarly, another 2020 study found that 70% of physicians agreed that videoconference consultations enabled access to care during the COVID-19 pandemic [20].

Limitations

Limited studies have looked into the barriers and facilitators of telemedicine in Riyadh, which may have limited the extent of our literature review. Convenience sampling was used in our study and was distributed only among physicians at KKUH in Riyadh, so it may not be representative of other healthcare facilities. Additionally, the questionnaire used in this study, although piloted, was not validated and was based on a literature review, which may lead to unavoidable biases.

## Conclusions

Telemedicine has an inevitable role in the future of health care due to its cost-effectiveness and potential to save time. Moreover, the use of telemedicine has considerably increased since the start of the COVID-19 pandemic. However, different specialties face a number of different barriers and facilitators. Furthermore, the potential of telemedicine implementation would depend on the specialty needs and work environment.

Our study showed that there are several obstacles that need to be addressed to improve telemedicine's role in providing care, especially technical difficulties. Thus, our study highlights the need for future studies to investigate potential ways to resolve this issue.

## Appendices

**Table 5:** Questionnaire: demographics & pattern of use

Demographics & Pattern Of Use
Age
21–30
31-40
41-50
51-60
> 60
Gender
Male
Female
Job Position
Intern
Resident
Specialist
Consultant
Specialty
Internal medicine
Surgery
Dermatology
Orthopedics
Emergency
Pediatrics
Family Medicine
Radiology
Obstetrics & Gynecology
Other
Nationality
Saudi
Non-Saudi
Have you ever used telemedicine in patient consultation?
Yes
No
When did you utilize telemedicine?
Before COVID-19
During COVID-19
Both
Which methods did you utilize?
Phone call
Video call
Email messages
Text messaging
Other
How often do you use Telemedicine?
Daily
Weekly
Monthly
Occasionally

**Table 6:** Questionnaire: perceived facilitators of telemedicine implementation

Perceived facilitators of telemedicine implementation
Telemedicine can lower unnecessary patient’s face-to-face appointments.
Strongly disagree
Disagree
Neutral
Agree
Strongly agree
Telemedicine is easily understood and used by physicians.
Strongly disagree
Disagree
Neutral
Agree
Strongly agree
Telemedicine is cost-efficient for the hospital.
Strongly disagree
Disagree
Neutral
Agree
Strongly agree
Telemedicine can increase patients' understanding and adherence to treatment plans.
Strongly disagree
Disagree
Neutral
Agree
Strongly agree
Telemedicine can ensure high standards of health care.
Strongly disagree
Disagree
Neutral
Agree
Strongly agree
Telemedicine enhances the accessibility to health care.
Strongly disagree
Disagree
Neutral
Agree
Strongly agree

**Table 7:** Questionnaire: perceived barriers of telemedicine implementation

Perceived barriers of telemedicine implementation
Telemedicine diagnosis is unreliable due to lack of physical examination.
Strongly disagree
Disagree
Neutral
Agree
Strongly agree
There is a lack of training in the use of telemedicine for physicians.
Strongly disagree
Disagree
Neutral
Agree
Strongly agree
Patient’s privacy is a barrier to the implementation of telemedicine.
Strongly disagree
Disagree
Neutral
Agree
Strongly agree
Physicians are resistant to changing from face-to-face consultations to virtual consultations
Strongly disagree
Disagree
Neutral
Agree
Strongly agree
Technical difficulties are a barrier to telemedicine implementation.
Strongly disagree
Disagree
Neutral
Agree
Strongly agree
Outdated hardware is a barrier to telemedicine.
Strongly disagree
Disagree
Neutral
Agree
Strongly agree
Difficulty in detecting non-verbal cues is a barrier to telemedicine.
Strongly disagree
Disagree
Neutral
Agree
Strongly agree
